# Feasibility Study on the Use of Fly Maggots (*Musca domestica*) as Carriers to Inhibit Shrimp White Spot Syndrome

**DOI:** 10.3390/life11080818

**Published:** 2021-08-11

**Authors:** Po-Yu Huang, Yi-Hsuan Huang, Jiann-Horng Leu, Li-Li Chen

**Affiliations:** 1 Centre of Excellence for the Oceans, National Taiwan Ocean University, No. 2, Pei-Ning Road, Keelung 20224, Taiwan; abcm1042@hotmail.com; 2 Institute of Marine Biology, National Taiwan Ocean University, No. 2, Pei-Ning Road, Keelung 20224, Taiwan; anita12140505@gmail.com (Y.-H.H.); jiholeu@ntou.edu.tw (J.-H.L.)

**Keywords:** vaccine, oral delivery system, maggot, immune response, white spot syndrome

## Abstract

The shrimp aquaculture industry has encountered many diseases that have caused significant losses, with the most serious being white spot syndrome (WSS). Until now, no cures, vaccines, or drugs have been found to counteract the WSS virus (WSSV). The purpose of this study was to develop an oral delivery system to transport recombinant proteinaceous antigens into shrimp. To evaluate the feasibility of the oral delivery system, we used white shrimp as the test species and maggots as protein carriers. The results indicated that the target protein was successfully preserved in the maggot, and the protein was detected in the gastrointestinal tract of the shrimp, showing that this oral delivery system could deliver the target protein to the shrimp intestine, where it was absorbed. In addition, the maggots were found to increase the total haemocyte count and phenoloxidase activity of the shrimp, and feeding shrimp rVP24-fed maggots significantly induced the expression of penaeidins 2. In the WSSV challenge, the survival rate of rVP24-fed maggots was approximately 43%. This study showed that maggots can be used as effective oral delivery systems for aquatic products and may provide a new method for aquatic vaccine delivery systems.

## 1. Introduction

Shrimp aquaculture brings crustaceans of substantial economic value and an important source of income in many countries worldwide. According to Food and Agriculture Organisation (FAO) statistics, in 2018, the farmed shrimp industry produced more than 9.4 million tons of product worth more than USD 69.3 billion (*Litopenaeus vannamei*, 52.9%; *Procambarus clarkia*, 18.2%; *Eriocheir sinensis*, 8.1%; *Penaeus monodon*, 8.0%; *Macrobrachium nipponense*, 2.5%; *Macrobrachium rosenbergii*, 2.5%; Other crustaceans, 7.8%). As with other high-density aquaculture species, shrimp are threatened by many pathogens, including acute hepatopancreas necrosis disease, Taura syndrome virus, and white spot syndrome virus (WSSV) [1]. WSSV is highly lethal to all shrimp life stages, and because 100% cumulative mortalities can be reached within 3–10 days under farming conditions, it results in massive economic losses worldwide. WSSV is the causative agent of white spot syndrome (WSS) disease, which has a wide host range, infecting all crustaceans. The disease is characterised by the appearance of white spots of 0.5 mm to 2.0 mm in diameter on the inside surfaces of the carapace, appendages, and the cuticle covering the abdominal segments. WSSV, the only member of the genus *Whispovirus*, which was assigned to a new family, *Nimaviridae*, is a large, enveloped, ellipsoid, and double-stranded DNA virus [2,3,4,5,6,7].

Until now, at least 58 structural proteins have been identified in WSSV, 30 of which are classified as envelope proteins. VP28 and VP26 are the most abundant, accounting for 60% of the envelope proteins. Envelope proteins play important roles in viral infection, such as the recognition of and attachment to receptors on the host cell surface [8,9,10,11,12,13]. Many studies showed envelope proteins interact with host proteins such as VP28/PmRab7 and VP187/β-integrin [9,14], and recently, the interactions between WSSV envelope proteins and host proteins were further confirmed. After interactions between nine structural proteins were identified, the ‘infectome’ concept was proposed [8]. A part of the infectome binds to chitin-binding protein, and VP24 connects it to the rest of the infectome [15].

Blocking the interactions between WSSV envelope proteins and host receptors could reduce viral infection burdens. Studies have found that envelope proteins, such as VP19, VP24, VP28, and VP53A, can inhibit WSSV infections [16,17,18,19], and many anti-WSSV strategies have used recombinant WSSV envelope proteins to block or induce the shrimp immune response to WSSV infection. Recombinant proteins have been examined and expressed in various systems, including viruses, yeast, *Escherichia coli*, baculovirus, and transgenic animals [20,21,22,23,24]. Although recombinant protein-expressing systems show effectiveness against WSSV in shrimp, the issues of efficiently transferring a recombinant protein or immune stimulant into shrimp intestines without damaging the protein structure, and translating research results into marketable technologies need to be addressed. Oral administration is a convenient, labour-saving vaccination method for aquaculture compared with injection or immersion and is less stressful to shrimp. 

Many insects are used as feed in aquaculture, including housefly maggots (*Musca domestica*), black soldier flies (*Hermetia llucens*), silkworm pupae (*Bombyx mori*), and mealworms (*Tenebrio molitoretc*) [25,26]. Housefly larvae, or maggots, are used for environmental and medical health reasons, such as recycling waste food and removing gangrene from wounds, as well as in the production of maggot meal. Maggot meal contains 39–61.4% crude protein, 12.5–21% lipids, and 5.8–8.2% crude fibre and is rich in phosphorus, trace elements, and B complex vitamins [27]. The biological value of maggot meal is equivalent to that of whole fish meal, and the larvae contain antibiotics and no anti-nutritional or toxic factors [28]. However, the mass commercial production of fly maggots for raising shrimp is still in the development stage. There is a need for innovations in technology to enhance maggot production and the application of maggot meal in shrimp aquaculture.

The purpose of this study was to determine the possibility of transporting recombinant WSSV protein into maggots and to investigate the protective effects against WSSV in shrimp. The data showed that the recombinant protein accumulated in a biologically embedded manner in the maggot, and the maggot protected the recombinant protein from enzymatic digestion during transport to the shrimp intestine, where it induced specific immune gene expression. The results from this study suggest that maggots are a potential oral vaccine system for shrimp aquaculture.

## 2. Materials and Methods

### 2.1. Experimental Animals

*Litopenaeus vannamei* shrimp (average ~5 ± 1 g) were purchased from southern Taiwan. Shrimps were selected for their vigour and hard-shell excellence and kept in 320 ppt sterilised seawater for 7 days at 26 ± 1 °C until the challenge experiment. Adult houseflies (*Musca domestica*) collected in proximity to the National Taiwan Ocean University were used to establish breeding colonies. Larva migration within the manure mass during development was assessed using clear plastic containers (H: 30 cm × W: 20 cm × L: 20 cm). A mixture of meat and water was provided ad libitum in open containers as oviposition substrate for the flies. The larvae were harvested before pupation at the second instar after approximately 2–4 days of growth and were rinsed in PBS buffer before being used in the following analysis.

### 2.2. Virus and Viral Inoculum

We used WSSV (GenBank Accession no. AF440570) isolated in 1994 in Taiwan from infected *Penaeus monodon* [7]. The haemolymph was collected from experimentally WSSV-infected shrimps *(L. vannamei*; mean weight: 15 g), diluted 1:4 with PBS, and frozen at −80℃. The shrimp were inoculated with the virus by feeding with infected shrimp meat, and the natural infection was monitored. The infected shrimp meat was prepared as follows: specific pathogen-free shrimp were treated with 100 μL WSSV (1.7 × 10^5^ copy number/ng) via intramuscular injection, and infected shrimp were collected 7 days into the virus challenge. Then, the shrimp meat was extracted, chopped, and mixed evenly, and 0.5 g of infected shrimp meat transferred to a new microcentrifuge tube and stored at −20 °C. The WSSV content of the infected shrimp meat was measured using the Innocreate Bioscience WSSV QD Kit (Innocreate Bioscience, New Taipei City, Taiwan) and estimated according to the relevant standards and formulas provided in the kit. The viral content was maintained at 10^5^ copies/μL.

### 2.3. Expression of Recombinant WSSV VP24 (rVP24) and Enhanced Green Fluorescent Protein in E. coli

The WSSV envelope protein VP24 was amplified from the genomic DNA of the WSSV T-1 strain with the primers VP24-F/VP24-R, and enhanced green fluorescent protein (EGFP) was amplified from the EGFP plasmid with the primers EGFP-F/EGFP-R (Table 1). The resultant recombinant plasmid pET28b-rVP24 and pET28b-EGFP was transformed into *E. coli* BL21 (DE3) strain. *E. coli* BL21 (DE3) cells were cultured in LB medium (10 g of tryptone, 5 g of yeast extract, 10 g of NaCl, and 1 L of distilled water) with 25 μg/mL kanamycin at 37 °C, and protein expression was induced with 1 mM isopropyl-β-D-thiogalactopyranoside at 30 °C for 8 h. The recombinant protein tagged with six consecutive histidines was purified with QIAexpressionist nickel-nitrilotriacetic acid metal-affinity chromatography (Qiagen, Hilden, Germany) according to the manufacturer’s recommendations. The resins were washed with buffer (pH 8.0) containing 50 mM sodium phosphate, 0.3 M sodium chloride, and 10 mM imidazole, and protein was eluted with buffer (pH 8.0) containing 50 mM sodium phosphate, 0.3 M sodium chloride, and 250 mM imidazole. The eluted protein was then concentrated using Amicon Ultra-15 centrifugal filters (Merck Millipore, Burlington, MA, USA) in PBS buffer and stored at 4 °C for further antiserum production.

### 2.4. Antisera Production

New Zealand white rabbits were used to develop polyclonal antisera against rVP24. In brief, the rabbits were hyperimmunised by injection with 250 μg protein emulsified in complete Freund’s adjuvant. Subsequent booster injections were carried out with 250 μg protein emulsified in incomplete Freund’s adjuvant. The antisera were collected after the antibody titre had peaked.

### 2.5. Evaluation of Protein Carrying Ability of Maggots

After the expression of the rVP24 was induced in *E. coli*, the culture was centrifuged for 2 min at 13,000 rpm and the supernatant discarded. The *E. coli* pellet was mixed with PBS buffer. Second-instar maggots were rinsed in PBS buffer, which was removed before the following analysis. The maggots (rVP24-fed maggots) were placed in 9 cm Petri dishes and fed the pET28b-rVP24 solution, and collected after 30, 45, 60, 75, 90, 105, and 120 min. After all maggots had ingested at least 30 μg of rVP24, they were washed in PBS buffer and drained. The maggot samples were freeze-dried using a lyophilisation system (Kingmech, New Taipei City, Taiwan) at −40 °C for 16 h before being stored in a humidity control box. Western blotting was used to evaluate the rVP24 protein degradation times in the maggot digestive tract. *E. coli* expressing EGFP was used as an indicator for the evaluation of the protein-carrying ability of the maggots. The recombinant plasmid pET28b-EGFP containing green fluorescent protein in the open reading frame was used as an enrichment indicator. The rEGFP-fed maggots were prepared using the same method described for as the rVP24-fed maggots. The location of the recombination protein in maggots and shrimp was observed using fluorescence microscopy (Olympus IX71 395 nm, Tokyo, Japan).

### 2.6. Evaluation of the Ability of The Maggot Vector to Deliver Protein to The Shrimp Digestive System

To study the delivery of the recombinant protein to the digestive system of *L. vannamei* shrimp, they were fed freeze-dried maggots containing *E. coli* transformed with rEGFP as an indicator, as previously described. Negative control shrimp were fed normal maggots. Shrimp weighing 10 ± 1 g were first used to stock an indoor 80-L aquarium and fed commercial feed twice daily (09:30 and 19:00) for 1 week to acclimate to the experimental conditions. At the end of the acclimation period, the shrimp were fed commercial shrimp feed in the morning and fly maggot feed in the evening. During the experimental period, the water temperature ranged from 26 ± 1 °C, and the photoperiod followed a 12:12 light: dark schedule. Shrimp from each group were randomly sampled each day of the feeding trial and anaesthetised by placing on ice, and the alimentary canal was removed. After rinsing with PBS, the stomach and intestines were separated by tissue scissors aseptically. The stomach and intestines were observed using fluorescence microscopy. 

### 2.7. SDS-Page

A discontinuous electrophoresis buffer system with 4% stacking gel and 12% resolving gel was used for protein separation. All samples were boiled for 10 min after the addition of sample loading buffer and subsequently electrophoresed at a voltage of 80 V for the stacking gel and 120 V for the resolving gel until the bromophenol blue reached the bottom of the gel. Protein bands were visualised by staining with Coomassie Brilliant Blue R-250. 

### 2.8. Western Blotting

For Western blotting analyses, proteins separated by SDS-PAGE were transferred onto a polyvinylidene difluoride membrane (Merck Millipore, Burlington, MA, USA) by semi-dry blotting. Membranes were blocked in 5% skim milk (Difco Laboratories, Sparks, MD, USA) in TBS (0.2 M NaCl and 50 mM Tris-HCl, pH 7.4). Immunodetection was performed by incubating the blot in rabbit anti-VP24 serum diluted 1:5000 in TBS with 5% skim milk for 1 h at room temperature. Subsequently, goat anti-rabbit IgG antibody conjugated with horseradish peroxidase (Sigma-Aldrich, St. Louis, MO, USA) was used at a concentration of 1:10,000, and detection was performed with Western Blot Chemiluminescence Reagent (NEN Life Sciences, Boston, MA, USA).

### 2.9. WSSV Testing by Conventional PCR

Screening of shrimp gill tissue to identify WSSV-positive samples was conducted using the IQ2000 WSSV PCR Kit (GeneReach, Taichung, Taiwan). DNA was extracted from the pleopods using the supplied DNA lysis buffer in accordance with the manufacturer’s instructions. WSSV-positive samples were graded as extremely light, light, moderate, or heavy using the banding pattern of PCR products, as recommended by the manufacturer.

### 2.10. Quantitative Real-Time PCR Assay

The WSSV QD Kit quantitative system (Innocreate Bioscience, New Taipei City, Taiwan) was used to quantify the absolute WSSV genomic DNA copy number in the pleopods collected from the WSSV-infected shrimp in the feeding study. Three pooled samples were prepared for each datapoint, with each pooled sample containing pleopods from three shrimps. The shrimp DNA and WSSV genomic DNA were then extracted using the DTAB/CTAB DNA extraction kit (GeneReach, Taichung, Taiwan). The samples were analysed on a real-time PCR system in accordance with the instructions provided in the WSSV QD Kit manual. The real-time PCR data were analysed using 7500 software (Applied Biosystems, Foster City, CA, USA). To calculate the results (copies/μL), the following equations were applied to convert the values into WSSV copies per nanogram of shrimp DNA:

Shrimp DNA = Shrimp DNA copy number/Shrimp index (10872)

Ratio of virus copy to shrimp DNA = WSSV DNA copy number/Shrimp DNA

To assess the reproducibility of the standard curve, standard reactions were performed three times independently, including duplications of each reaction. The data were analysed by using the statistical program and presented as the mean ± SD

### 2.11. Feeding Trial

To compare the difference in the WSSV load and immune parameters of shrimp after feeding with the maggot vector, we used the IQ2000 WSSV Kit to select WSSV-positive shrimp and graded their positivity from extremely light to moderate. All the shrimp (10 ± 1 g) were randomly allocated to four groups of triplicates in 12 tanks. The groups were classified according to different diets (commercial shrimp feed, pET28b-fed maggot vector, rVP24-fed maggot vector, and normal maggot). The preparation of all maggot diets followed a previously described Materials and Method 2.5: the maggot was freeze-dried 1 h after feeding on different *E. coli*; the pET28b-fed maggot vector fed pET28b to the plasmid negative control to check the plasmid could not produce protection; the rVP24-fed maggots fed rVP24 to the experimental group; normal maggots did not feed any *E. coli* to the negative group. The shrimp were fed commercial shrimp feed in the morning and maggot feed in the evening for 15 days. During the feeding trial period, three shrimp from each group were randomly sampled on days 0, 3, 6, 9, 12, and 15.

### 2.12. Total Hhaemocyte Count

To conduct the total haemocyte count (THC), three shrimp from each group were randomly sampled. The pooled samples were prepared for each data point, with each pooled sample containing pleopods from three shrimps. Haemolymph (300 μL) was withdrawn from the ventral sinus of each shrimp and mixed with anticoagulant buffer (anticoagulant, 27 mM sodium citrate, 336 mM NaCl, 115 mM glucose, and 9 mM EDTA, pH 7.0) at a 1:1 ratio. The haemocytes were counted using haemocytometer and a phase-contrast microscope (Nikon, Tokyo, Japan)

### 2.13. Phenoloxidase Activity

Phenoloxidase (PO) activity was measured following a previously described method. Anticoagulant buffer was mixed with the haemocytes at a ratio of 1:1, and the cells were collected by centrifugation at 1000× *g* for 20 min at 4 °C. The cell pellet was resuspended in 1 mL cacodylate citrate buffer (10 mM sodium cacodylate, 0.2 M NaCl, 10 mM trisodium citrate, pH7.0) and centrifuged again. The supernatant was removed, and the pellet was resuspended in 200 μL cacodylate buffer (10 mM sodium cacodylate, 0.2 M NaCl, 10 mM CaCl_2_, 0.26 M MgCl_2_, pH 7.0). The aliquot was equally divided into two tubes, with one tube used for measuring total PO activity and the other for measuring background PO activity. Cacodylate buffer (100 μL) was added to the sample tube to measure total PO activity, and 100 μL 1% SDS buffer was added to the sample tube to measure background PO activity. After 10 min, 50 μL of 0.3% L-dihydroxyphenylalanine were added to the tubes for 5 min. We used a spectrophotometer at 490 nm to measure PO activity, and the amount of inactive PO was calculated as the total available PO minus PO activity before SDS treatment.

### 2.14. RNA Extraction and Real Time-PCR Analysis

Shrimp gill tissues were homogenised in 1 mL of TRIzol reagent (Thermo Fisher Scientific Inc. Waltham, MA, USA) and then subjected to 2-propanol extraction and ethanol precipitation of total RNA in accordance with the manufacturer’s recommendations. The total RNA was centrifuged in 75% ethanol at 14,000× *g* for 30 min at room temperature, and the pellet was dissolved in Diethylpyrocarbonate (DEPC)-treated water and quantified by spectrophotometry. After RNA extraction, 1 μg of total RNA was used for cDNA synthesis using HiScript I Reverse Transcriptase (BIONOVAS, Toronto, ON, Canada) with an oligo (dT) 18 primer according to the manufacturer’s protocol. The synthesis condition of cDNA was set at 65 °C for 5 min, 42 °C for 60 min, and 70 °C for 15 min. Real-time PCR was performed using the Applied Biosystem 7500 Real-Time PCR System (Applied Biosystems, Waltham, MA, USA) on a TOptical thermocycler (Analytik Jena AG, Jena, Germany). The gene expression of penaiedin 2 (*PEN2*), crustin, superoxidase dismutase (*SOD*), clotting protein (*CP*), *Litopenaeus vannamei* toll receptor (*LvToll*), and elongation factor-1α (*EF-1α*) genes were measured using the primers listed in Table 1. The real-time PCR reaction contained 1 μL of cDNA template, 10 μL of 2 × qPCRBIO syGreen Master Mix, and 0.8 μL each of the forward and reverse primers (10 pmol/uL). The amplification conditions were an initial denaturation at 95 °C for 10 min, followed by 40 cycles at 95 °C for 15 s and 60 °C at 60 s. The melting curve and cooling were performed in the last step of the PCR. The expression levels of the target gene were normalised to *EF-1α*, a shrimp housekeeping gene. The fold change in relative gene expression compared with the control group was determined by the standard 2^−^^△△Ct^ method. The changes were analysed using an unpaired sample t-test. Statistical significance was accepted at *p* < 0.05, and high significance was accepted at *p* < 0.01. All data were expressed as mean ± standard deviation (mean ± SD).

### 2.15. In Vivo Neutralisation Assay

According to the Guide for Animal Use Protocol of the Institutional Animal Care and Use Committee (IACUC) of National Taiwan Ocean University, ethical approval was not required. The shrimp were acclimatised in the laboratory for about 1 week before the experiment. The experimental shrimp were then further divided into four groups, with three replicates of 20 shrimp in each group. The shrimp were fed as follows: the positive control group 1 was fed with commercial shrimp feed, the plasmid negative control group 2 was fed pET28b-fed maggots, group 3 was fed normal maggots, and group 4 was fed rVP24-fed maggots. During the neutralisation assay period, the diet and water conditions were the same as in the above feeding trial, and the WSSV inoculum step was initiated after 9 days. During the experimental period, the shrimp survival rates of each group were recorded every day. The cumulative survival rates were calculated and subjected to a paired sample t-test, and differences were considered significant at *p* < 0.05.

## 3. Results

### 3.1. Optimum Conditions for Incorporating Recombinant E. coli into Maggots

**Figure 1:**
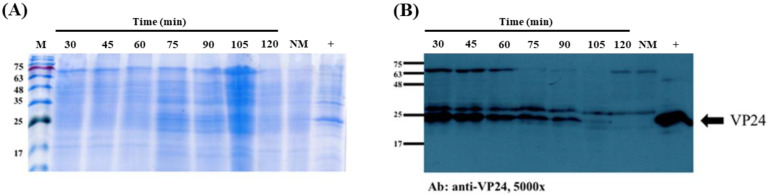
Digestion time of recombinant VP24 (rVP24) protein in maggots. (**A**) Coomassie brilliant blue-stained 12% SDS-PAGE gel. M: marker, NM: normal maggot, +: rVP24 *E. coli* lysate. (**B**) rVP24 protein on Western blot was recognised by the specific anti-VP24 antiserum.

**Figure 2:**
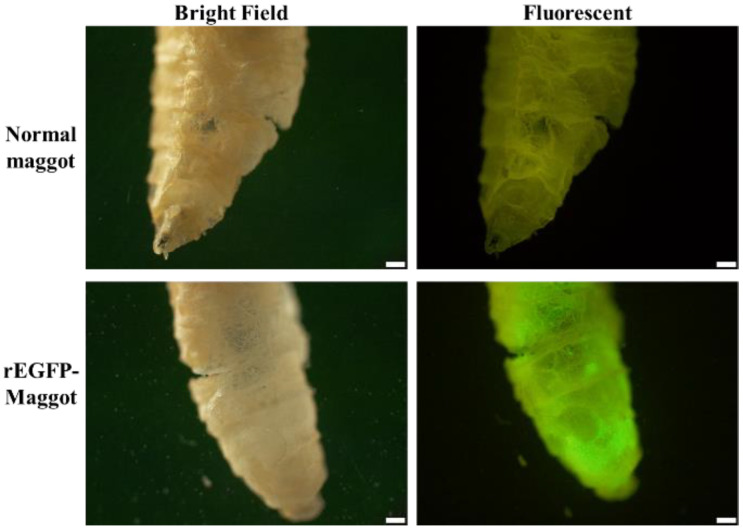
EGFP signal in fly maggots from control and experimental groups. The fly maggots were fed rEGFP and freeze-dried, and the fluorescent signal was detected. Control: normal maggot. Scale bar: 0.4 mm.

The maximum time that the recombinant protein remains intact in the maggots is an important factor. Thus, the aim of this analysis was to evaluate the digestion time of the recombinant protein in the maggot. The second-instar fly maggots were fed the *E. coli* solution containing rVP24. Using Western blotting, we observed the presence of rVP24 from 30 to 105 min, before it disappeared by 120 min. The data showed the recombinant protein was maintained in the maggot for at least 105 min (Figure 1). After feeding the maggots *E. coli* solution containing rEGFP for 1 h, the EGFP signal was observed in the maggots via fluorescence microscopy, and no signal was seen in the negative control normal maggots (Figure 2).

### 3.2. Delivery of Recombinant Protein to The Shrimp Digestive system

**Figure 3:**
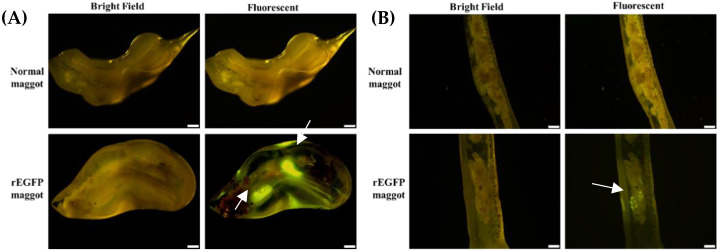
rEGFP signal (white arrow) in shrimp gastrointestinal tract from normal maggot groups and rEGFP-fed maggot groups. (**A**) stomach (**B**) intestines. Fluorescent signal was seen in the group fed EGFP-fed maggots. Scale bar: 100 μm.

**Figure 4:**
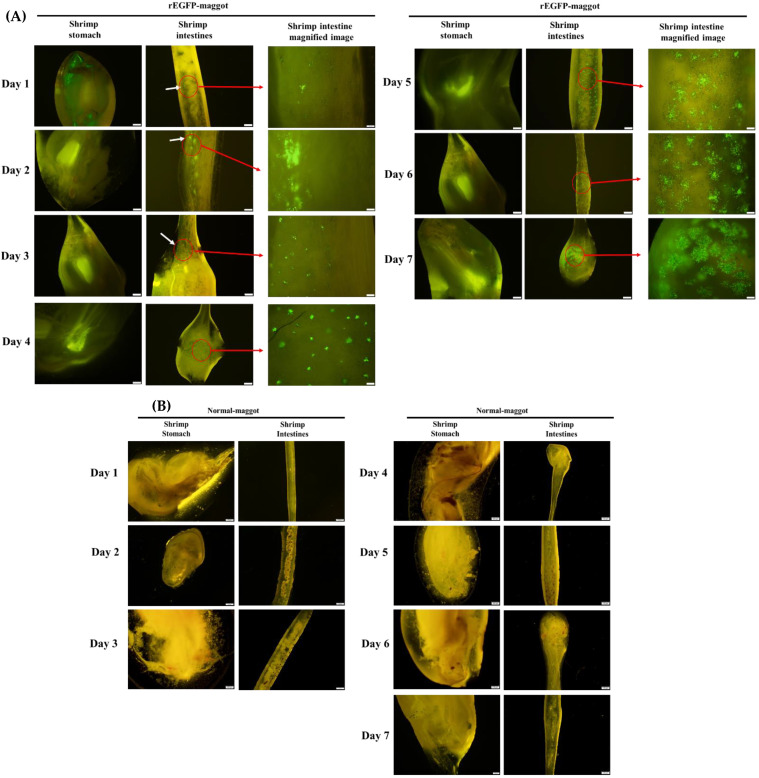
Fluorescent signal in shrimp gastrointestinal tract during continuous feeding. (**A**) The fluorescence (white arrow) in the shrimp stomach and intestine increased with feeding days (1~7 days). (**B**) No fluorescent signal was observed during the feeding trial with normal maggots in the shrimp stomach or intestine. Scale bar: 100 μm. Magnified image scale bar: 20 μm.

**Figure 5:**
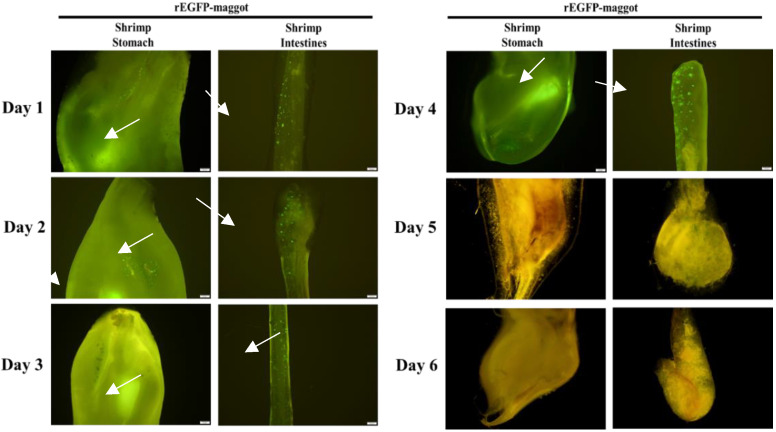
Fluorescent signal in shrimp gastrointestinal tract after feeding ceased. rEGFP (white arrow) remained in the gastrointestinal tract for 4 days. No fluorescent signal was observed during the feeding trial with normal maggots in the shrimp stomach or intestine (data not shown). Scale bar: 100 μm.

Delivery of the recombinant protein to the digestive system of the shrimp was demonstrated directly by fluorescent imaging. When maggots containing *E. coli* expressing EGFP protein (rEGFP-fed maggots) were fed to the shrimp, the fluorescent EGFP signal was seen in the gastrointestinal tract within 3 h, and no fluorescence was observed in stomach and intestine of shrimp fed normal maggots (Figure 3A,B). We then ascertained whether the target recombinant protein accumulated in the digestive system of the shrimp when they were continuously fed the maggot vector. During the feeding trial period, one shrimp was randomly sampled every day to observe the gastrointestinal tract. Fluorescence was seen to increase in the shrimp stomach and intestine during the feeding period. The fluorescence was detected in the stomach at all data points. On the 1st day, a weak fluorescence signal was detected in the shrimp intestine wall. On the 2nd day, more fluorescence was detected in the shrimp intestine wall. On the 3rd day, the fluorescence’s situation was similar to the 2nd day, but the distribution was wider. A significant increase in the fluorescence range can be observed on the intestinal wall from the 4th to the 7th day (Figure 4A). No fluorescent signal was observed during the feeding trial with normal maggots in the shrimp stomach or intestine (Figure 4B). Furthermore, we observed the decrease in fluorescence after we stopped providing the rEGFP-fed maggot in order to understand how long the rEGFP can remain in the gastrointestinal tract. When we stopped feeding the rEGFP-fed maggot, we still could observe fluorescence in the stomach and intestine wall from day 1 to 4. The rEGFP fluorescent signal was maintained in the gastrointestinal tract of the shrimp for 4 days, and there was no fluorescent signal after the 5th day (Figure 5).

### 3.3. In Vivo Test of WSSV Genome Copy Numbers, THC, and PO Activity

**Figure 6:**
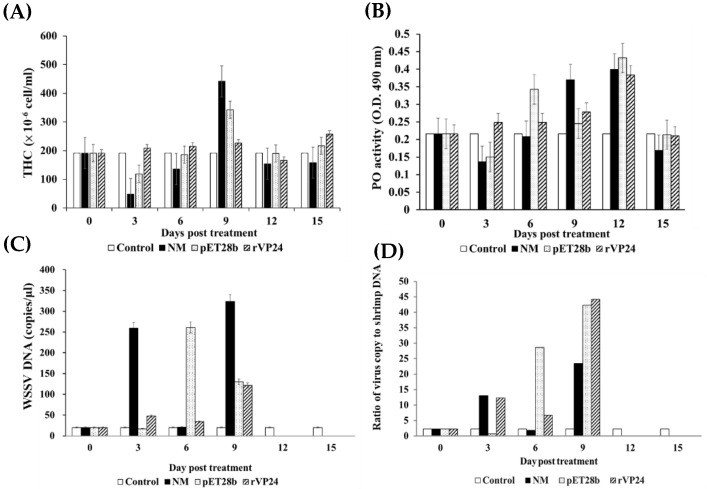
(**A**) Total hemocyte counts (THC), (**B**) phenoloxidase (PO) activity, (**C**) WSSV DNA copy number, and (**D**) ratio of virus copy to shrimp DNA in haemocytes from *Litopenaeus vannamei* fed commercial shrimp feed (Control), feed normal maggots (NM), pET28b-fed maggots (pET28b), or rVP24-fed maggots (rVP24) during 15-day feeding trial. Data are expressed as mean ± SEM (*n* = 3).

The changes observed in the total haemocyte count (THC) in the haemolymph of WSSV-positive shrimp are shown in Figure 6A. Compared with the control group, the THC in the normal maggot and pET28b-fed maggot groups changed by a relatively large extent, with significantly lower THC than the control group on days 3 and 6, but higher THC on day 9. However, the rVP24-fed maggot group maintained a relatively stable THC that was slightly higher than that of the control group. The change in phenoloxidase (PO) activity in the haemolymph was similar to that of the THC: PO activity was lower in the normal maggot group than the control group on day 3, but then rose on days 6, 9, and 12, peaking on day 12, and gradually stabilising on day 15. The PO activity of the pET28b-fed maggot group was lower than that of the control group on day 3, but rose on days 6 and 12, peaked on day 12, and gradually stabilised on day 15 (Figure 6B). During the feeding trial, all WSSV-positive shrimps survived. The WSSV genome copy number peaked on day 9 in all groups, but no WSSV was detected in the shrimp on days 12 and 15 (Figure 6C,D).

### 3.4. Expression of Innate Immune-Related Genes

**Figure 7:**
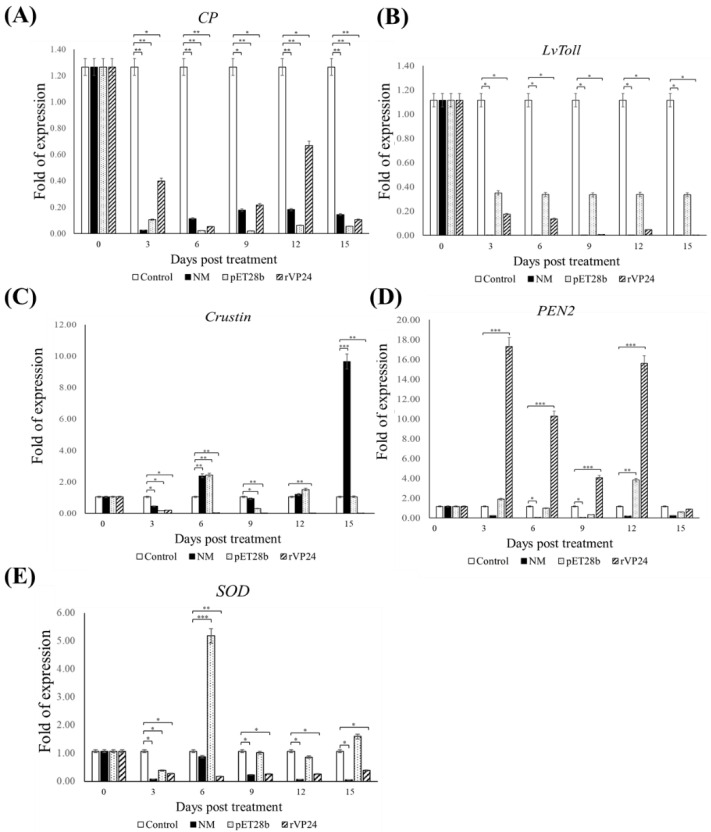
Expression of (**A**) *CP*, (**B**) *LvToll*, (**C**) crustin (**D**), *PEN2*, and (**E**) *SOD* in haemocytes from *Litopenaeus vannamei*-fed commercial shrimp feed (Control), normal maggots, (NM), pET28b-fed maggots (pET28b), or rVP24-fed maggots (rVP24) during the 15-day feeding trial. Data are expressed as mean ± SEM (*n* = 3), and significant differences from the control group are indicated by asterisks. Significant differences (*p* < 0.05) between the compared groups are indicated with asterisk (⁕). Highly significant differences (*p* < 0.005) of gene expression level between the compared groups are indicated with double asterisks (⁕⁕). Triple asterisks (⁕⁕⁕) indicated greater significant difference (*p* < 0.0005).

We performed gene expression analysis to evaluate the transcription levels of immune genes in the gills of *L. vannamei* fed the maggot feed and control diets. All maggot-fed groups showed a significant downregulation of clotting protein (*CP)* and *Litopenaeus vannamei* toll receptor *(LvToll)* gene expression (Figure 7A,B). The expression of crustin was upregulated in the normal maggot group on day 6 and significantly upregulated on day 15, but this returned to a level similar to the control group on the other days. We also noted the rVP24 maggots showed significant downregulation of crustin expression at every time point (Figure 7C). Significantly upregulated expression of *PEN2* was seen in the rVP24 maggots on days 3, 6, 9, and 12, returning to a level similar to the control group on day 15 (Figure 7D). The *SOD* gene expression levels were significantly higher in the pET28b maggot group only on day 6 and returned to a level similar to the control group on the other days. The other groups showed downregulated expression at all time points (Figure 7E).

### 3.5. WSSV Neutralisation In Vivo by rVP24-fed Maggots

**Figure 8:**
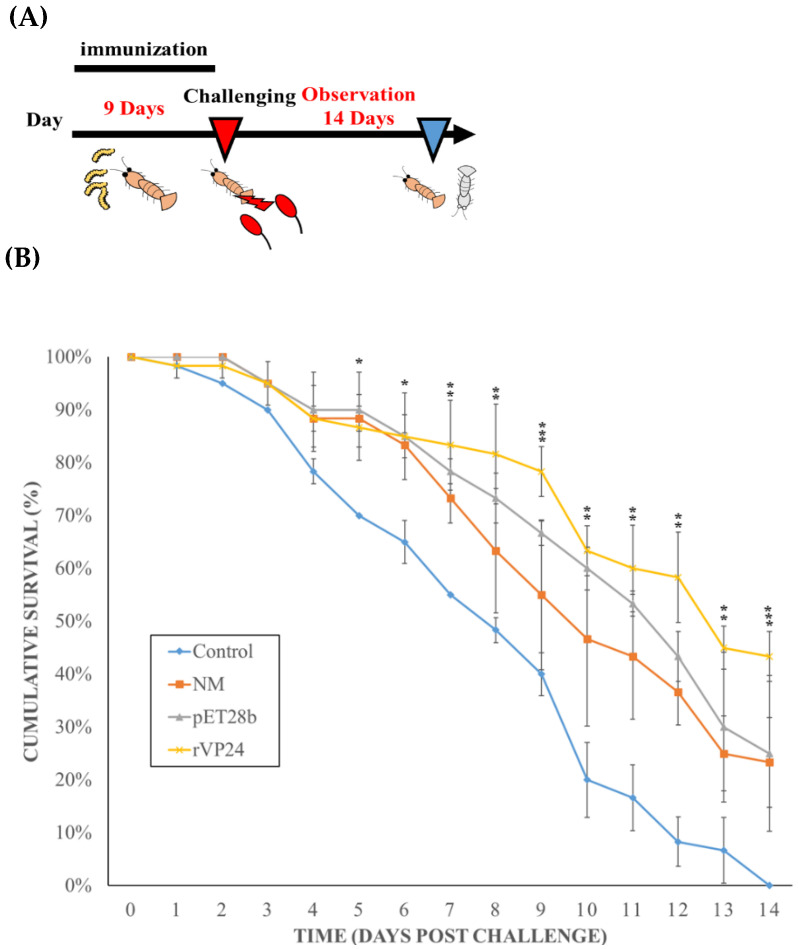
Evaluation of the rVP24-fed maggots for protective effects against WSSV. (**A**) Time schedule of shrimp immunisation, WSSV challenge, and observation. The immunisation strategies for four different groups (commercial shrimp feed, pET28b-fed maggots, normal maggots, rVP24-fed maggots) are shown. In brief, shrimp were immune at day 0 and challenged with WSSV via fed WSSV-shrimp meat 9 days afterward. (**B**) In vivo neutralisation assay. In vivo neutralisation of WSSV infection in shrimp using maggot oral vaccine system (*n* = 20). Group 1, positive control fed commercial shrimp feed (♦); group 2, plasmid negative control fed pET28b-fed maggots (▲); group 3 fed normal maggots (■); group 4 fed rVP24-fed maggots (×). In all groups, the virion inoculum was equivalent to 10^5^ copies. The line marked with an asterisk (⁕) is significantly higher than that of the control groups. Highly significant differences (*p* < 0.005) of gene expression level between the compared groups are indicated with double asterisks (⁕⁕). Triple asterisks (⁕⁕⁕) indicated greater significant difference (*p* < 0.0005).

The protection conferred by the maggot vector was evaluated through an experimental WSSV challenge. Shrimp were infected with the virus by feeding them WSSV-infect shrimp meat, and the survival rates were evaluated for 2 weeks. Compared with the control group, there was a significant difference from the 5th day (*p* < 0.05), and the greater significant difference (*p* < 0.0005) appeared from the 8th day and remained until the end of the experiment; the rVP24-fed maggot group maintained the greater significant difference from 6th to 14th days; the normal maggot group and pET28b-fed maggot group maintained the highly significant differences (*p* < 0.005) from 6th to 14th days. There are some significant differences between the normal maggot group, pET28b-fed maggot group, and rVP24-fed maggot group, but the overall trend is not significantly different. The final survival rate for the rVP24-fed maggot group was 43.3%, that of the normal maggot group was 23.3%, and that of the pET28b-fed maggot group was 25% (Figure 8).

## 4. Discussion

Maggots have always been considered to have excellent properties as feed additives or fishmeal because they have multiple antimicrobial peptides and a large quantity of high-quality protein. The antibacterial compounds found in maggots are capable of lysing over 90% of Gram-positive and Gram-negative bacteria, including *Pseudomonas aeruginosa*, *Klebsiella pneumoniae*, and methicillin-resistant *Staphylococcus aureus*, within 15 min by changing the membrane potential of the bacteria [29,30]. However, as maggot production is still limited and the optimum percentage of maggots to add to shrimp feed has not yet been determined, maggot feed still needs to be developed [31,32,33]. In this study, we demonstrated that a recombinant WSSV protein vector delivered using maggots as an oral vaccine system induced a protective immune response in shrimp. The improved efficacy of this oral vaccine system over other oral vaccine systems may be due to several factors: First, using the natural food of shrimp as a carrier facilitated the uptake of the recombinant protein; second, the *E. coli* cell wall and the maggot intestine protected the recombinant protein from gastrointestinal degradation and enabled its delivery and accumulation in the shrimp gastrointestinal system; third, the antimicrobial peptides in the maggot or incorporated *E. coli* could produce an innate immune response.

The recombinant WSSV protein used was VP24, which plays a key role in the WSSV infectome, and EGFP was chosen as indicator for the evaluation of the protein-carrying ability of the maggot. Maggots were fed *E. coli* that were induced to express VP24 and collected every 15 min for a total of 2 h. As shown in Figure 1, the VP24 antibody signal did not weaken until 2 h after feeding, indicating that rVP24 persisted in the maggots for 105 min. This result is consistent with that found by other research teams, who showed that bacteria are digested by fly maggots within approximately 1 to 1.5 h [34]. The Coomassie blue staining showed the presence of a major 75 kDa band that was likely to be the insect’s haemcyanins, and the Western blot assay detected specific antibody signals at approximately 75 kDa and 30 kDa in both the normal maggot group and rVP24-fed maggot group. Compared to the rVP24 *E. coli* liquid, these were considered to be non-specific signals caused by the proteins in the maggot. These non-specific signals will be used as the basis for subsequent judgments. To directly observe the capacity of the maggot to carry recombinant proteins, the maggots were fed rEGFP and freeze-dried. Through an inverted fluorescent microscope, we observed green fluorescence in the maggot after feeding, indicating that the maggots had the ability to carry and preserve the recombinant protein (Figure 2). Based on the results of the maggot digestion experiments, the maggot will be freeze-dried 1 h after feeding on *E. coli* in subsequent experiments.

After feeding rEGFP-fed maggots to the shrimp, green fluorescent signals were observed in the stomach (Figure 3A) and intestines (Figure 3B), which proved that the maggot can protect the recombinant protein from degradation so that it reaches the shrimp’s gastrointestinal tract. Moreover, rEGFP may accumulate in the shrimp intestines when the rEGFP-fed maggots are fed continuously (Figure 4). Once the rEGFP-fed maggot diet was stopped, the green fluorescent signal was maintained in the shrimp’s gastrointestinal tract for 4 days until it disappeared on day 5 (Figure 5). Continued feeding of rEGFP-fed maggots was shown to cause the recombinant protein to accumulate in the shrimp gastrointestinal tract, where it was maintained until its digestion.

During the process of aquaculture, shrimp are easily infected with WSSV, and the survivors become asymptomatic carriers. We selected WSSV-positive shrimp to observe changes in the THC, PO activity, and WSSV copy number after they were fed with maggots. In the maggot feeding trial, WSSV was still detectable on days 3, 6, and 9 following ingestion, but after day 12, no WSSV was detected in any group (Figure 6). Previous studies found that THC levels and PO activity declined with prolonged infection. We also found this phenomenon in the normal maggot and pET28b-fed maggot groups on day 3. As feeding days progressed, THC and PO activity gradually increased in the normal maggot and pET28b-fed maggot groups, while the WSSV copy number decreased, returning to the original level on the 15th day. This may be because the maggots stimulated THC and PO activity and cleared the WSSV. THC and PO activity in the rVP24-fed maggot group was maintained at a higher level than those in other groups, but the number of WSSV copies did not change significantly, which is similar to the situation in shrimp supplemented with immune stimulants after infection. Hence, supplementing with rVP24 not only maintained THC levels and PO activity from being inhibited by WSSV but also helped shrimp to reduce the WSSV copy number.

We chose genes relevant to different immune pathways, *SOD* of the proPO system, *CP* of the coagulation system, *PEN2* and crustin of the antimicrobial peptide system, and Toll-like receptor (*LvToll*), and observed whether the maggots regulated the expression of these genes to resist WSSV (Figure 7). The gene expression of *CP* and *LvToll* was downregulated in all groups. Although crustin and *SOD* expression increased significantly at some time points, there was no overall upregulation. We noticed the gene expression of *PEN2* was significantly upregulated, and shrimp fed a diet of rVP24-fed maggots showed 4 to 17 times greater *PEN2* expression than the control groups. According to Xiao et al.’s research, WSSV infection activates the Toll and IMD (NF-κB-related) signalling pathways that induce the production of penaeidins, including BigPEN, PEN2, PEN3, and PEN4. Specifically, PEN2 interferes with the ability of the receptor-binding protein VP24 to bind to the host receptor LvpIgR, thereby blocking viral entry into the target cells [35]. The real-time PCR data showed that the rVP24-fed maggot carried rVP24 to the digestive tract of the shrimp, where it successfully induced a specific immune gene response.

After confirming that maggots can deliver the recombinant protein to the shrimp intestine, we used an in vivo neutralisation assay to test whether the maggot delivery system can reduce the mortality rate after WSSV infection. Based on the results of the previous experiments, the shrimp were fed with commercial shrimp feed or difference maggots for 9 days before the in vivo neutralisation experiment to ensure sufficient protection and reduce the remaining WSSV in the body. Then, the shrimp group were fed on the 9th day to start the WSSV inoculation. The results (Figure 8) showed that the positive control group (group 1 given commercial shrimp feed) showed a 0% survival rate on the 14th day after infection. The initial mortality and final survival rates of the normal maggots (group 2) and pET28b-fed maggots (group 3) were 23.3% and 25%, respectively. This could be due to the compound in the maggots stimulating the host defence system. Group 4, which was fed the rVP24-fed maggots, showed obvious protection, as the survival rate was 43.3%. Therefore, we concluded that the rVP24-fed maggots delayed or neutralised the WSSV infection. The in vivo neutralisation assay results were similar to previous VP24 in vivo neutralisation results [36]. In the first 10 days, the survival rate of the maggot-fed groups was maintained at 50%, then gradually decreased, which was possibly because the maggot feed replaced evening meals in our experiments, causing the shrimps to become nutrient-deprived for some time. The maggots may have to be used as an additional functional feed instead of as a replacement for fish meal.

Many studies have found that envelope proteins, including VP19, VP24, VP28, and VP53A, can inhibit WSSV infection. However, the most efficient method of delivering these preventive proteins to the shrimp gastrointestinal tract was unclear. To achieve efficient vaccination, the ability of the carrier to store the target protein, the ease of operation, and the palatability of the carrier to the shrimp must be considered. In this study, we successfully showed that the target protein persisted in the maggot and was stable at room temperature, and the treated maggot successfully delivered the target protein to the shrimp gastrointestinal tract. When we fed the shrimp the treated maggots, shrimp THC levels and PO activity levels were maintained, specific immunity gene expression was regulated, and the survival rate after WSSV infection was improved. 

After hatching, the first-instar maggot is roughly 2 and 5 mm long, the second-instar maggot grows to around 10 mm and the third-instar maggot grows to between 15 mm and 20 mm. The method used in this study can carry recombinant protein to maggots of each instar, which will be adjusted according to the size of the farmed animals. This study established a preliminary method for using maggots, which are palatable to shrimp, as a potential oral vaccine system for shrimp aquaculture. It is hoped that, in the future, this system can be applied to disease prevention or treatment in other aquatic animals.

## 5. Conclusions

Both direct and indirect evidence in this study indicate the feasibility of using fly maggots as a protein carrier for aquatic products. We successfully confirmed that the tar-get protein persists in the maggot and is stable at room temperature, and the treated mag-got can successfully deliver the target protein to the gastrointestinal tract of the shrimp. After feeding the treated maggots to the shrimp, the THC level and the PO activity level of the shrimp were maintained, the specific immune gene expression was regulated, and the survival rate after WSSV challenge was improved. The method used in this study can carry recombinant protein to maggots of each instar, and adjust which instar to use ac-cording to the size of the mouthparts of the farmed animals. This study established a preliminary method for using maggots, which are palatable to shrimp, as a potential oral vaccine system for shrimp aquaculture. It is hoped that, in the future, this system can be extended to disease prevention or treatment of other aquatic animals.

## Figures and Tables

**Table 1:** Primers used in this study.

Gene	Primer	Sequence 5′→3′	Purpose
VP24	VP24-F	5′-CGGGATCCGACCAACATAGAACTTAAC-3′	Recombinant protein
VP24	VP24-R	5′-GAGAGAATTCTTTTTCCCCAACCTTAAAC-3′	Recombinant protein
EGFP	EGFP-F	5′-ATCCGAATTCGATGGTGAGCAAGGGCGAGGAG-3′	Recombinant protein
EGFP	EGFP-R	5′-CTGAGCTCCTTGTACAGCTCGTCCATGCCGA-3′	Recombinant protein
Penaiedin2	PEN2-F	5′-TCGTGGTCTGCCTGGTCTT-3′	RT-qPCR
Penaiedin2	PEN2-R	5′-CAGGTCTGAACGGTGGTCTT-3′	RT-qPCR
Crustin	crustin -F	5′-GAGGGTCAAGCCTACTGCTG-3′	RT-qPCR
Crustin	crustin -R	5′-ACTTATCGAGGCCAGCACAC-3′	RT-qPCR
Superoxidase dismutase	*SOD*-F	5′-ATCCACCACACAAAGCATCA-′	RT-qPCR
Superoxidase dismutase	*SOD*-R	5′-AGCTCTCGTCAATGGCTTGT-3′	RT-qPCR
Clotting protein	CP-F	5′-TCTTTGCGCAGTTGGTGATC-3′	RT-qPCR
Clotting protein	CP-R	5′-TGAGGTGACCGAGTGCAAAA-3′	RT-qPCR
LvToll receptor	LvToll-F	5′-ATGTGCGTGCGGATACATTA-3′	RT-qPCR
LvToll receptor	LvToll-R	5′-GGGTGTTGGATGTCGAGAGT-3′	RT-qPCR
Elongation factor 1-α	EF1α-F	5′-TGCCCTGGACAACATCGAGC-3′	RT-qPCR
Elongation factor 1-α	EF1α-R	5′-CGGGCACTGTTCCAATACCT-3′	RT-qPCR

## Data Availability

Data reported in this paper can be obtained from the authors upon request.
